# Treatment of Acute Otitis Media with Inner Ear Involvement in Adults

**DOI:** 10.3390/jcm12247590

**Published:** 2023-12-09

**Authors:** Soner Dogan, Alexander M. Huber, Christof Roosli

**Affiliations:** Department of Otorhinolaryngology, Head and Neck Surgery, University Hospital Zurich, University of Zurich, 8091 Zurich, Switzerland; soner.dogan@usz.ch (S.D.);

**Keywords:** acute otitis media (AOM), inner ear complication, inner ear involvement/disease (IED), antrotomy

## Abstract

Inner ear involvement (IED) is a rare local complication of the very common acute otitis media (AOM). The most beneficial treatment for IED remains a matter of debate. The aim of this study is to analyze different treatment modalities based on hearing outcomes to contribute to the discussion of therapy for IED in AOM. This retrospective study includes 112 adult patients diagnosed with AOM with IED between 2000 and 2020. Patients either received conservative (systemic antibiotic and systemic steroid therapy), interventional (conservative plus myringotomy and tympanic tube) or operative (interventional plus antrotomy) treatment. Pre- and post-treatment pure tone audiometry was performed. The hearing outcome was compared, and hearing recovery was analyzed based on modified Siegel’s criteria. The pre-treatment pure tone average (PTA) was significantly (*p* < 0.05) higher in the operative group than in the other groups. All treatment modalities led to a significant hearing improvement (*p* < 0.001). The pre- and post-treatment hearing loss was predominantly observed in high frequencies 2–4 kHz. The operative group showed the highest rate of complete hearing recovery. While all treatment modalities led to a significant improvement in hearing, the operative group showed the most beneficial hearing results in patients with high pre-treatment hearing loss. It remains to be shown if the findings in patients with high pre-treatment hearing loss can be generalized to patients with mild or moderate pre-treatment hearing loss.

## 1. Introduction

Acute otitis media (AOM) is a very common disease with a global incidence rate of 5.3 cases per 1000 person-years [1]. Although generally self-limiting, AOM is the ear disease with the most acute complications, local or intracranial [2,3,4]. One of the local complications is an inner ear involvement (IED) with sensorineural hearing loss (SNHL) and/or vestibular symptoms. The incidence rates for IED/SNHL vary from 2.2% to 67% depending on the form of otitis media [5,6,7,8].

Acute otitis media can be categorized as a mainly pediatric health care issue. Hence, data on the treatment of the adult patient is scarcer. AOM is defined as an acute onset of characteristic otologic symptoms, presence of middle ear effusion and other signs of acute middle ear inflammation [9]. This inflammation is mainly caused by bacterial infections subsequent to a viral infection of the upper airways [10]. During this inflammation, the thickness and permeability of the round window membrane may be altered and inflammatory agents, i.e., cytokines and bacterial exotoxins, may diffuse to the inner ear, causing IED [11,12]. It may affect the basal turn of the cochlea, resulting in temporary or permanent sensorineural hearing loss [13]. These labyrinthine effects do not always appear in the acute phase of the infection and may be observed with a delay of up to 3 to 4 weeks [14]. Inflammatory mediators released in the middle ear cavity may play a role in the pathogenesis. Damage to the inner ear is described to arise from the passage of toxic substances from the middle ear through the round window membrane into the cochlea [15]. This may cause a perilymphatic inflammatory reaction, starting in the basal turn of the cochlea and damaging the inner and outer hair cells. Another path to spread the inflammation is described through the mastoid antrum, which manifests clinically with an abscess of the mastoid, possibly with bone destruction [16]. Therefore, a practical approach may include antibiotic therapy (bacterial infection), corticosteroid therapy (anti-inflammatory effect) and reduction of inflammatory agents by removing them from the middle ear via myringotomy and ventilation tubes and/or the mastoid antrum via an antrotomy (operative opening of the mastoid antrum). 

Unfortunately, data on standardized treatment for IED in AOM is rare and the benefit of antrotomy is not well-investigated. There is some evidence that the hearing outcome with primary treatment (steroids and antibiotics) with an additional antrotomy results in a better hearing outcome than primary therapy alone in patients with IED in otitis media with effusion and subacute mastoiditis after viral infection [14]. It may be similar in patients with IED and AOM. Hence, the aim of our study is to retrospectively analyze the hearing outcome of patients with IED in AOM, with regard to different treatment strategies such as systemic antibiotic therapy, systemic steroid therapy and myringotomy with or without antrotomy.

## 2. Materials and Methods

A retrospective, single-center data analysis at the University Hospital of Zurich was performed, including all adult patients between 2000 and 2020 that were diagnosed with AOM with IED. An IED was defined as bone conduction threshold of >15 dB in at least two frequencies between 250 and 4000 Hz with normal hearing on the not affected contralateral side and/or vestibular symptoms. Patients with suppurative labyrinthitis were excluded because of the more severe and irreversible course of the disease.

All enrolled patients were tested with pre- and post-treatment pure tone audiometry. A pure tone audiogram was obtained with 5 dB steps at frequencies of 0.125, 0.25, 0.5, 1, 2, 4 and 8 kHz for air conduction and 0.25, 0.5, 1, 2 and 4 kHz for bone conduction for all patients on both sides delivered by an Interacoustics Equinox 2.0 audiometer (InteracousticsVR, Middelfart, Denmark) in a sound-isolated booth. Pure tone averages (PTA) were set as mean threshold values in bone conduction (BC) from 0.5, 1, 2 and 4 kHz. 

According to the received treatment, patients were assigned to either the conservative, interventional or operative group. Conservative treatment solely included antibiotics and systemic steroid therapy. Unless prior side effects or allergies were documented, the main antibiotic therapy consisted of amoxicillin/clavulanic acid for 7–14 days. It was administered either orally or intravenously. Systemic steroid therapy consisted of dexamethasone 40 mg/day (250 mg) for 3 days followed by 10 mg/day (62.5 mg) for another 3 days or the equivalent dosage of prednisolone (in brackets) [17,18,19,20]. Topical therapy with ciprofloxacin/hydrocortisone was not administered regularly. 

Interventional treatment was defined as conservative treatment with additional myringotomy and/or insertion of ventilation tube. Depending on the patient, inserted tubes varied in size and material. Mainly Tuebingen-type ventilation tubes (Bess GmbH, Berlin, Germany) made of titanium were inserted. These procedures were performed in local anesthesia in the outpatient department. 

Lastly, operative treatment consisted of interventional treatment with additional antrotomy. The antrotomy is a surgical procedure wherein the mastoid antrum is opened. The surgery was performed as follows. Postauricular skin incision is made, followed by dissection of a periosteal flap. After displaying the mastoid plane and the outer ear canal, drilling of the mastoid bone commences by skeletonizing the dura of the middle cranial fossa until the lateral semicircular canal and the incus body are identified. As a final step, the middle ear is rinsed through the opened antrum [21]. 

The post-treatment hearing outcome was classified according to modified Siegel’s criteria [22] as either complete recovery, partial recovery or no recovery (Table 1). 

Statistical analysis was performed with the Statistical Package for Social Sciences (SPSS, version 29.0). Paired and unpaired *t*-tests were used to compare pre- and post-treatment bone conduction hearing thresholds within each group and between the groups. *p*-values < 0.05 were considered statistically significant.

## 3. Results

### 3.1. Demographics and General Findings

A total of 112 patients were enrolled in this study. According to the treatment modality, they were divided into the conservative group (N = 24, 21.4%), the interventional group (N = 35, 31.3%) and the operative group (N = 53, 47.3%). The majority in each group and overall were female patients, at a percentage of 63%. There was no significant difference in mean PTA between male and female patients. 

The most common symptoms stated by the patients were subjective hearing loss (112/112, 100%), otalgia (111/112, 99%), otorrhea (61/112, 54%), tinnitus (57/112, 51%) and vertigo (34/112). The mean and range of PTA value sorted by the respective symptom was as follows. Hearing loss (33.5 dB, 6.25–71.25 dB), otalgia (33.6 dB, 6.25–71.25 dB), otorrhea (33.3 dB, 6.25–71.25 dB), tinnitus (32.7 dB, 6.25–67.5 dB) and vertigo (39.5 dB, 8.75–67.5 dB). By comparison of the mean PTA of patients with or without a specific symptom, a significantly higher mean PTA (*p*-value: 0.003) was observed in vertigo patients. Additionally, no other symptom showed a significant decrease or increase in the PTA. Detailed demographics and symptoms according to the groups are displayed in (Table 2).

### 3.2. Hearing Outcomes and Recovery Rates

**Figure 1:**
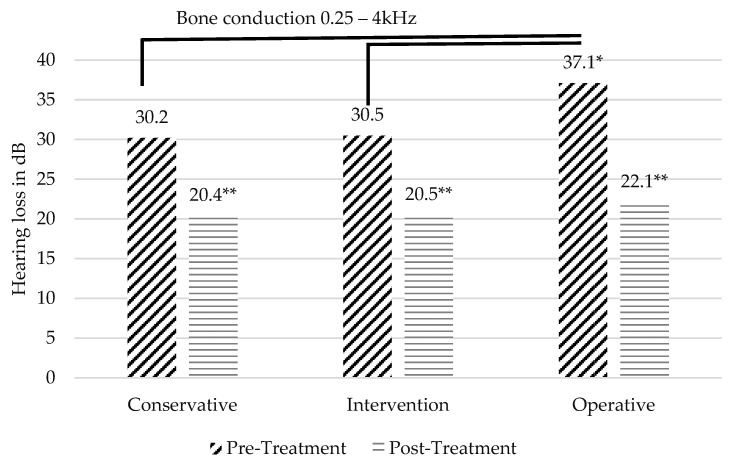
BC pure tone averages (PTA) pre- and post-treatment. Significant improvement was observed within all groups (** *p* < 0.001). Significantly higher threshold pre-treatment was observed in operative group (* *p* < 0.05).

The mean audiometry follow-up period was 5½ months (166 days, ranging from 5 to 1888 days). The mean follow-up was 39 days in the conservative group, 205 days in the interventional group and 243 days in the operative group. The pre-treatment PTA of the conservative group was 30.2 dB, 30.5 dB in the interventional group and 37.1 dB in the operative group. The pre-treatment PTA in the operative group was significantly higher compared to the conservative (*p*-value: 0.04) and compared to the interventional group (*p*-value: 0.03) (Figure 1). Between the conservative and the interventional group, there was no significant difference.

Post-treatment, a significant improvement in hearing thresholds was observed in all three groups (*p* < 0.001). The hearing improvement was highest in the operative group, which improved from 37.1 to 22.1 dB, followed by the interventional group, which improved from 30.5 to 20.5 dB and the conservative group, which improved from 30.2 to 20.4 dB. 

**Figure 2:**
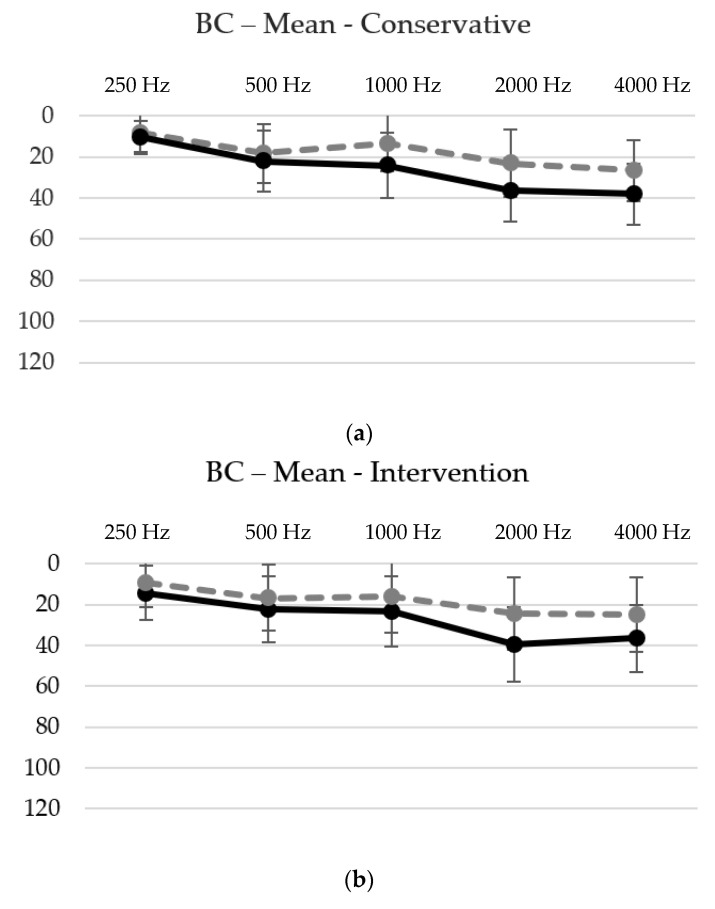

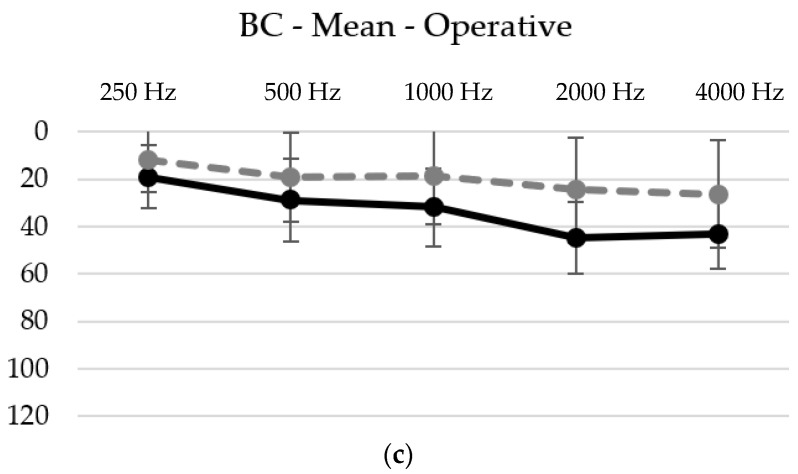
(**a**–**c**) BC thresholds pre-treatment (solid line) and post-treatment (dotted line) for each group ((**a**) conservative group, (**b**) intervention group and (**c**) operative group) and frequency with the corresponding standard deviation. The thresholds increase significantly in the higher frequencies, with a peak at either 2 kHz or 4 kHz. See also Table 3.

In further analysis, the hearing loss was predominantly observed in the higher frequencies before and after treatment, as demonstrated in Figure 2a–c and Table 3. In all three groups, the following pattern was observed. 

Firstly, the mean hearing loss was at its lowest at 0.25 kHz, with values of 10.6 dB in the conservative group, 14.4 dB in the interventional group and 19.1 dB in the operative group. Secondly, there was a highly significant (*p*-values < 0.001) increase in the hearing threshold from 0.25 kHz to 0.5 kHz. Thirdly, there was no statistical difference between 0.5 kHz and 1 kHz, except for in the operative group, where an additional significant (*p*-value < 0.05) hearing loss was observed. Fourthly, another highly significant (*p*-values < 0.001) increase in the hearing threshold was observed between 1 kHz and 2 kHz. Lastly, a similar hearing threshold with no significant difference was observed between 2 kHz and 4 kHz. 

The highest mean hearing thresholds were observed at 4 kHz with 38.1 dB in the conservative, at 2 kHz with 39.4 dB in the interventional and at 2 kHz with 44.7 dB in the operative group. 

**Figure 3:**
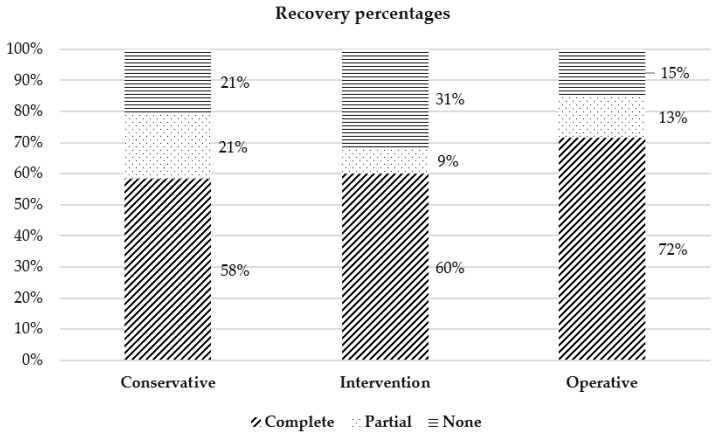
Hearing recovery rates for each group based on modified Siegel’s criteria (*p* > 0.05). Diagonal pattern indicates complete recovery, dotted indicates partial and horizontal indicates no recovery.

Within every group, a high rate of complete recovery (58% in the conservative, 60% in the interventional and 72% in the operative group) was achieved. In between the groups, a tendency for a higher rate of complete recovery in the operative group was observed, although it was not statistically significant (Figure 3).

## 4. Discussion

### 4.1. Mechanisms of Pathology

A possible complication of AOM is IED. However, little is known about the prevalence of IED in AOM and the mechanism of IED in AOM remains unclear. It is hypothesized that inflammatory agents or mediators transmigrate or diffuse through the round window membrane or stapedial ligament. It was shown in an animal experiment that interleukin 2 (IL-2) provoked a sensorineural hearing loss in the high frequencies when instilled in the middle ear cavity near the round window, reaching the maximum concentration of IL-2 after 18 h and maximum hearing loss after 5 to 7 days. This emphasizes the indirect or mediator effect of IL-2 [23]. On the contrary, an animal study on pneumolysin, a pneumococcal protein, has shown a dose-dependent toxic effect within minutes and, in addition, at sublytic concentration induction of pro-inflammatory mediators when perfused into the cochlea [24]. It is a matter of discussion if a surgical intervention leads to clearing the middle ear space from either viral/bacterial toxins or inflammatory mediators or if another mechanism occurs. 

### 4.2. Demographics and General Findings

Generally, the male gender is described to be associated with a higher risk of acute otitis media and its recurrent forms [2,3,9,25,26]. In the present study, the majority of the patients were female, at a percentage of 63%. This might be due to the fact that most epidemiologic data come from descriptions in pediatric cohorts, not specified in uncomplicated or complicated forms of AOM, while the present study was limited to adult patients with AOM with IED. 

Contrary to the difference in genders, the mean age of all groups was very similar, ranging from 43.8 years in the interventional group to 48.3 years in the conservative group, improving the comparability of the respective groups. 

Regarding the clinical findings, the majority of the patients had subjective hearing loss and ear pain. There was also a high percentage of patients with tinnitus (51%) and vertigo (32%). IED may lead to vestibular symptoms ranging from mild dizziness to immobilizing rotatory vertigo, clinically demonstrating itself with various patterns of nystagmus, either irritative, paretic or direction-changing, depending on the pathologic mechanisms and time delay to the initial examination after onset [27,28]. In the current study, no uniform records of nystagmus or apparative diagnostics were performed. Instead, vertigo was assessed from the patient’s history. The prevalence of vertigo was slightly lower compared to the findings in the literature [29]. However, the incidence of vertigo was higher in the interventional group (37%) and operative group (39.5%) compared to the conservative group. New peripheral vertigo may be a factor for a more invasive therapy because it can be considered a sign of pathologic affection of the labyrinth.

Notably, none of the patients with vestibular symptoms were treated conservatively. The significantly higher mean PTA (see Table 2) in combination with the vertigo symptom in itself may have been perceived as a more severe case, leading to more invasive interventional or operative therapies. 

### 4.3. Hearing Outcome and Recovery

An IED was defined as >15 dB in two frequencies between 250 and 4000 Hz. There is a great variability in the definition of IED in the literature, ranging from 15 to 30 dB [30]. The intention was to use a small inner ear component to also analyze the outcome of patients with a small IED. 

The hearing loss was higher in the high frequencies with a statistically significant increase between 250 and 500 Hz, as well as between 1000 and 2000 Hz in all treatment groups. Additionally, there was a significant increase between 500 and 1000 Hz in the operative group (Table 3). This finding indicates structural damage of the cochlea that is more severe in the basal turn. It has been shown that inflammatory mediators are present in the middle ear cavity during inflammation. The round window is permeable to many substances, and the cells in the vicinity of the round window are more likely to be affected [15].

First of all, our data show a significant improvement in bone conduction thresholds in all three groups, no matter which therapy modality they received. Therefore, it is consistent with the previously reported data of Park et al. and Song et al. [5,31]. They both showed a significant improvement in hearing thresholds after antibiotic therapy, systemic steroid therapy and myringotomy with or without ventilation tube insertion. There is no exact previous data on the effect of postauricular antrotomy. Wilhelm et al. [14] investigated a similar but more extensive approach, where some patients with acute otitis with effusion or subacute mastoiditis were treated with a complete mastoidectomy and compared to a conservative treatment. The conclusion was that patients experienced a better hearing outcome when primary surgical treatment was performed. Accordingly, in the present study, the highest improvement was observed precisely after the antrotomy. Both surgical treatments appear to have a beneficial effect, though ranging from more temperate antrotomy to more extensive mastoidectomy, it is yet to be determined how aggressive the surgery has to be. 

An explanation from a pathophysiological standpoint might be the direct opening and rinsing of the middle ear, thereby reducing the inflammatory toxins passing through the round window to the inner ear [32,33,34,35]. However, it was not objectively investigated if inflammatory toxins were reduced. Further, it is unclear what the toxic threshold of these toxins is. 

Additionally, the higher improvement may be partially based on the higher pre-treatment pure tone average and not due to the therapy modality itself. 

From a different perspective, looking at the recovery rates, the highest percentage of complete recovery was observed in the operative group, at a rate of 72%. Based on previous data on sudden idiopathic hearing loss, it is known that the higher the sudden hearing loss is, the lower the probability of recovery becomes [36]. As shown in Table 2, in the modified Siegel’s criteria, complete recovery is not dependent on the pure tone average gain, but solely on the final pure tone average. Therefore, the higher recovery rate in the operative group is not simply explicable based on a higher pre-treatment hearing loss. In contrast, the higher rate of no recovery (31%) in the interventional group stands out, but with no obvious explanation so far. 

Nevertheless, the differences in recovery rates are not significant, maybe due to the yet insufficient sample sizes. Additionally, it is not clear if the findings in patients with high pre-treatment hearing loss can be generalized to patients with mild or moderate pre-treatment hearing loss. It remains to be seen if these observed tendencies become significant with further included patients. 

### 4.4. Limitations

A weakness of the present study is the selection bias. The patients were not randomly assigned to a group. Thereby, the number of patients in each group varied, causing varying powers of each group. Depending on the severity of the pre-treatment symptoms, the different treatment options were discussed individually. The treatment decision was therefore partly dependent on the responsible physician and his/her previous experience. Correspondingly, there is also a bias caused by the experience of the performing surgeon. The latter is kept to a minimum with standardized surgical steps and especially with the last checkpoint (rinsing of the middle ear via the opened antrum, thereby verifying the success of the operation). Further, it can not be excluded that an asymmetric hearing loss was present before the AOM in some patients.

A second weakness concerns the audiometry follow-up period. Since this is a retrospective study, there were no exact follow-up schedules. Some patients recovered very quickly from the IED and did not require a follow-up, others were lost for follow-up after 3 months. As mentioned above, the follow-up period was significantly lower for the conservative group and the highest in the operative group. A recall system for patients undergoing surgical intervention may have contributed to the differences in the follow-up period between the groups. 

On average, follow-up was 5–6 months, but some patients recovered even after 1 year. In order to ensure comparability, the recovery rates were addressed. Based on the recovery, patients with complete recovery had a mean follow-up of 182 days, patients with partial recovery had 120 days and those with no recovery had 246 days. These numbers indicate that there is no linear correlation between the degree of recovery and the follow-up length. Retrospectively, it is unknown what the driving factors for concluding the follow-ups were or what confounding factors may have caused this difference. Clearly defined follow-up schedules and endpoints (i.e., when a therapy might be concluded ahead of schedule) may facilitate future analysis and possible correlations between hearing recovery and follow-up length.

## 5. Conclusions

The hearing loss, and therefore the hearing recovery, was mainly observed in the higher frequencies, from 2 kHz onwards. The presence of vertigo was accompanied by significantly higher pretreatment hearing loss. 

All three therapy modalities led to significant hearing recovery.

The antrotomy represents a valid alternative, especially beneficial in patients with high pre-treatment hearing loss. It also demonstrated higher complete recovery rates, even in more severe cases.

The latter remains to be statistically verified in further investigations.

## Figures and Tables

**Table 1:** Modified Siegel’s criteria for hearing recovery outcomes. Slight improvement (final hearing level 46–75 dB) was included in Partial recovery *. Non-serviceable ear (final hearing level > 90 dB) was included in No improvement **.

Complete Recovery	Partial Recovery *	No Improvement **
Final hearing level/PTA ≤ 25 dB	More than 15 dB hearing gain and final hearing level/PTA 26–75 dB	Less than 15 dB hearing gain or final hearing level ≥ 76 dB

**Table 2:** Demographic factors and symptoms in pre-treatment medical history according to the respective group. Significantly higher mean PTA was observed in patients with vertigo, * *p* < 0.05.

	Conservative Group	Interventional Group	Operative Group	Total	Mean PTA
Patients					
N	24 (21.4%)	35 (31.3%)	53 (47.3%)	112 (100%)	33.5
Gender					
Male	9 (38%)	13 (37%)	19 (36%)	41 (37%)	33.7
Female	15 (62%)	22 (63%)	34 (64%)	71 (63%)	33.5
Age					
Mean (years)	48.3	43.8	45.5	45.6	
Range	26–79	16–66	19–85	16–85	
Symptoms					
Hearing loss	24	35	53	112	33.5
Otalgia	23	35	53	111	33.6
Otorrhea	13	16	32	61	33.3
Tinnitus	13	20	24	57	32.7
Vertigo	0	13	21	34	39.5 *

**Table 3:** Mean pretreatment hearing thresholds for each frequency from 0.25 kHz to 4 kHz for the corresponding group. The *p*-values underneath display the statistical significance to the next higher frequency. * *p*-values < 0.05 are considered significant.

	0.25 kHz	0.5 kHz	1 kHz	2 kHz	4 kHz
Conservative group	10.6 dB	22.1 dB	24.2 dB	36.3 dB	38.1 dB
*p*-values	0.000074 *	0.333	0.000007 *	0.493	
Interventional group	14.4 dB	22.4 dB	23.4 dB	39.4 dB	36.6 dB
*p*-values	0.000023 *	0.576	0.000001 *	0.247	
Operative group	19.1 dB	28.9 dB	31.9 dB	44.7 dB	43.0 dB
*p*-values	1.4526 × 10^−8^ *	0.021 *	1.0063 × 10^−10^ *	0.276	

## Data Availability

Available upon request.
